# Reducing high calorie snack food in young adults: a role for social norms and health based messages

**DOI:** 10.1186/1479-5868-10-73

**Published:** 2013-06-05

**Authors:** Eric Robinson, Ellis Harris, Jason Thomas, Paul Aveyard, Suzanne Higgs

**Affiliations:** 1 University of Liverpool, Psychological Sciences, Liverpool, UK; 2 University of Birmingham, School of Psychology, Birmingham, UK; 3 University of Oxford, Primary Care Health Sciences, Oxford, UK

**Keywords:** Social norms, Health messages, Junk food

## Abstract

**Background:**

Consumption of high calorie junk foods has increased recently, especially among young adults and higher intake may cause weight gain. There is a need to develop public health approaches to motivate people to reduce their intake of junk food.

**Objective:**

To assess the effect of health and social norm messages on high calorie snack food intake (a type of junk food) as a function of usual intake of junk food.

**Design:**

In a between-subjects design, 129 young adults (45 men and 84 women, mean age = 22.4 years, SD = 4.5) were assigned to one of three conditions: 1) a social norm condition, in which participants saw a message about the junk food eating habits of others; 2) a health condition, in which participants saw a message outlining the health benefits of reducing junk food consumption and; 3) a control condition, in which participants saw a non-food related message. After exposure to the poster messages, participants consumed a snack and the choice and amount of snack food consumed was examined covertly. We also examined whether usual intake of junk food moderated the effect of message type on high calorie snack food intake.

**Results:**

The amount of high calorie snack food consumed was significantly lower in both the health and the social norm message condition compared with the control message condition (36% and 28%, both p < 0.05). There was no significant difference in snack food or energy intake between the health and social norm message conditions. There was no evidence that the effect of the messages depended upon usual consumption of junk food.

**Conclusions:**

Messages about the health effects of junk food and social normative messages about intake of junk food can motivate people to reduce their consumption of high calorie snack food.

## Introduction

There has been a sharp increase in the availability and consumption of junk food in recent years and this may have contributed to rising rates of obesity [1,2]. Junk foods are defined as items that are high in energy content, fat and/or sugar and low in nutrients [3]. Consumption of junk food is of concern among young adults because they are a demographic identified as high consumers of junk food [4,5]. In one study, junk food intake was shown to account for 20% of variance in weight gain amongst young adults during a 3 month period [3].

There is mixed evidence that nutritional education and point of choice nutritional labelling encourage choice of ‘healthier’ foods over higher calorie ‘unhealthy’ options, such as junk food [6,7]. A large number of healthy eating approaches have been centred around increasing fruit and vegetable consumption [8,9] and public health messages about the health benefits of eating fruit and vegetables have had some success in increasing consumption [10,11]. The types of messages that motivate people to reduce junk food intake have received less attention. There are distinct differences between increasing fruit and vegetable intake and limiting junk food intake in terms of both the behavior (adding to the diet vs. limiting) and the foods (junk foods are more palatable). Thus, there is a need to develop an evidence base for message types that will motivate people to reduce their intake of high calorie junk foods.

In young adults, intake of high calorie snack food has been reported to be influenced strongly by perceived social norms, as the extent to which people believe others are consuming large or small amounts of high calorie snack food affects intake [12] (although see Salvy et al. [13] for other types of social influence on eating behaviour). Experimental studies have shown that participants eat greater amounts of high calorie snack food if they believe that previous participants in the study have eaten a large amount of snacks than if they believe the other participants have eaten a small amount [12,14]. These early studies made use of a ‘remote confederate’ design, whereby participants learn about previous participants either consuming small or large amounts of food. These findings are of interest, as participants eat alone and are therefore likely to be changing their behaviour as a result of the information they are exposed to, not just because they want to make a good impression or be liked by a dining companion. Similar effects have been observed in children, whereby beliefs about what other children have been eating have been shown to influence how much food both healthy weight and overweight children eat [15]. Another interesting observation is that being led to believe others have eaten very little food produces just as strong an effect on food intake as does eating with another person who has been instructed to eat very little [16]. Thus, it appears that social norms exert a substantial influence on food intake. Similar effects have also been reported on food choice. One study [17] found that if participants believe the norm is to select a high calorie snack food, they are more likely to make similar food selections. Finally, outside of the laboratory, beliefs about snack amounts consumed by peers are a significant predictor of snack food intake [18,19].

There is preliminary evidence that social norm messages can improve the healthiness of dietary choices [20,21]. An intervention study by Stok and colleagues [20] found that repeated exposure to social norms about fruit intake resulted in participants self-reporting increased fruit consumption. In a recent study we found that a social norm message was more effective than a health message at increasing the actual consumption of fruit and vegetables in a laboratory test meal [22]. The effect was moderated by participants’ usual intake of fruit and vegetables, whereby usually low consumers increased their intake, but usually high consumers did not. We hypothesised that this was because the low consumers felt motivated to change their behavior to adhere to the social norm message, while the high consumers were already doing so.

The use of social norm messages to motivate reductions in junk food consumption has not been examined. A novel hypothesis is that messages outlining that other people limit their junk food intake (a social norm message) may motivate individuals to reduce the amount of junk food they eat. As people are motivated to conform to social group norms [23], we might expect that high consumers of junk food would be more susceptible to norm messages. This is because high consumers of junk food are likely to perceive that they are out of line with the presented norm and adjust their behaviour accordingly, whereas low consumers of junk food will already be in line with the norm and so will be less likely to change their behaviour [23].

In the present laboratory study, we examined whether a health message and a social norm message about limiting junk food intake would motivate people to reduce their intake of high calorie snack food (a type of junk food). We exposed young adults to a message on a poster and observed their later food choices and consumption of both high calorie snack food and fruit and vegetables. By offering a choice of foods we were able to assess whether messages reduced junk food intake and whether this would also cause a corresponding reduction in total amount of energy consumed when alternative foods were available in their place. In line with research on healthy eating promotion [10,11], we hypothesised that participants in the health message condition would consume less high calorie snack food intake than participants in the no intervention control message condition. Based on our recent findings regarding the effect of social norm messages on fruit and vegetable consumption [22] we hypothesised that the social norms message about junk food intake may have a larger effect than the health message. We also predicted that the effect of message type may be moderated by usual junk food intake, whereby high consumers of junk food would be most influenced by the social norms message.

## Method

### Design

A 3 × 2 between-subjects design was used, with factors: message type (social norm/health /control) and usual junk food intake (low consumers/high consumers). Prior to taking part in what they believed was a mood and eating study, participants evaluated a poster containing a social norm, health or control message. Participants later completed mock mood measures before selecting and consuming a snack in a naturalistic setting.

### Participants

Sample size was determined using power calculations (GPower 3.1) on data from two recent studies that compared the use of social norm and health messages on fruit and vegetable intake in our study population [19]. At 80% power, α = 0.05, f = 0.28, a sample size of 127 participants was estimated to detect significant main and interaction effects. We aimed to recruit slightly above this number to account for any participants providing incomplete data sets.

One-hundred and twenty nine adults (45 men and 86 women) with a mean age of 22.4 years (SD = 4.5) were recruited through online and campus advertisement. The adverts suggested the study was about the effect that eating has on mood. Participation was in exchange for £5 Sterling or course credit. Participants were required to abstain from eating during the two hours prior to their session and could not have any food allergies. All participants that responded to advertisement were eligible. Recruitment took place between July 2012 and August 2012 at the University of Birmingham. The study was conducted according to the guidelines laid down in the Declaration of Helsinki and all procedures were approved by the University of Birmingham Research Ethics Committee. Participants gave written informed consent. We randomized participants through a simple random allocation sequence using researchrandomizer.org.

### Messages

Prior to arrival participants were randomly assigned to one of the three conditions. In the social norm and health conditions participants viewed a poster containing images of junk food (a hamburger, fries, soda, candy wrappers) and a message defining junk food: ‘junk food is high calorie food with low nutritional value’. The posters only differed in the content of a message in the middle of the poster. In the social norm condition this read ‘Students eat less junk food than you might realise. Most students limit how much junk food they are eating to 1 or less than 1 serving a day.* based on a 2012 study. This statistic was derived from a pilot survey study that we conducted with 40 students. The pilot study was an internet survey conducted shortly before data collection for the main study. Participants answered questions similar to the self-report measures described in the section ‘Measurements’. The aim was to formulate a norm message based on junk food intake in the target population.

In the health condition the message read ‘Reducing junk food intake is good for your health. Limiting junk food to 1 or less than 1 serving a day is part of a healthy diet. * based on a 2012 study’. In the control condition the poster was of a similar word length to the two other conditions, but the message emphasised the importance of preparing in advance for exams. The images were of textbooks. There was no reference to junk food in the poster.

### Food

The snack buffet consisted of 6 common food items in the UK. Three of these were high calorie snack foods; chocolate chip cookies (481 kcal/100 g), ready salted crisps (537 kcal /100 g) and chocolate finger biscuits (515 kcal/100 g). We selected these foods because participants in our pilot study reported that they ate these foods often and also that they were widely perceived as ‘junk food’. The fruit and vegetable items were carrot sticks (64 kcal/100 g), sliced apple (53 kcal/100 g) and grapes (64 kcal/100 g). All foods were purchased from Sainsbury’s Ltd (United Kingdom) and presented on individual plates. The participants were not made aware that we were examining food choice.

### Measurements

*Baseline hunger:* To assess baseline hunger the participants used a 10 cm line scale ‘how hungry are you right now? (mark with an x)’ with anchors ‘not at all’ and ‘extremely’. *Mock mood questionnaire:* To corroborate the cover story, the participants rated various moods (e.g. how *happy, tired, hungry, anxious, alert, angry, stressed* are you?) on separate 10 cm line scales, with anchors ‘not at all’ and ‘extremely’. *Cognitive restraint scale*: To assess dieting tendencies, all participants completed the cognitive restraint scale of the Three Factor Eating Questionnaire [24]. *Usual junk food intake measure:* To assess usual intake of junk food of participants we included two items. The first was a guided one day dietary recall measure [25]. Participants were asked to ‘think back carefully, working from when you woke up, please list all of the food items you ate yesterday’. Participants were then asked how many of the recalled items were junk food. We opted for participant classification (rather than a researcher coding this), as our hypothesis was that it would be participants’ *perceptions* of how much junk food they eat that would moderate the influence of a norm message. The second measure was of usual consumption. Participants were asked to indicate how many servings of junk food they normally ate a day. We did not provide explicit instruction about the types of food they should class as ‘junk food’. *Manipulation check:* To check whether participants classed the high calorie foods we included in the buffet as ‘junk food’ we asked participants to list the buffet items they classed as junk food. To check participants had correctly read the content of the poster, we asked participants to write down the message content.

### Procedure

Experimental sessions took place in the School of Psychology, University of Birmingham, UK. The sessions took place between 10 am-12 pm and 2-5 pm on weekdays. The researcher greeted the participant and explained they would be taking part in a mood study. The researcher also explained that another research group had developed some posters and because the mood study would not take long, it would be appreciated if the participant could provide some feedback on a poster first. After signing for consent for the poster study and providing demographic information, the participant was seated alone in a room and provided with the poster, which they rated for clarity and the extent to which they understood the messages (using a five point likert scale response format). The participant was then taken to another room to start the mood study and the researcher explained the cover story; the effect of eating on mood. The participant signed for consent, completed the baseline hunger measure and then the mock mood questionnaire. The participant was taken to a kitchen area where a naturalistic snack buffet was set out. After being provided with a plate, the participant was instructed to select whatever they wanted. Food selection and consumption occurred alone after which the researcher returned and gave the participant another mock mood questionnaire (to back up the cover story) and the cognitive restraint scale to complete. The participant was then asked to write down the aims of the study before having their height and weight measured using a stadiometer and digital scales to allow calculation of body mass index (BMI, kg/metres^2^). Finally, the participant completed the usual junk food intake measures and the manipulation checks. Participants were thanked and debriefed. The buffet foods were then weighed to calculate selections and any food selected but not consumed was also weighed.

### Strategy of analysis

We planned one way ANOVAs on baseline variables: hunger; BMI; restraint; and age, in order to check whether there measures differed according to condition. If one or more variables were found not to be balanced across conditions and correlated with the main dependent variable (grams of high calorie snack food consumed), we controlled for this using covariates in subsequent analyses.

Our main planned analysis strategy was to use a between-subjects 2×3 ANOVA to compare the independent and interaction effect of condition and usual intake on our main dependent measure: grams of high calorie snack food consumed. We planned the same strategy to examine the effect of condition and usual intake of junk food on total kcals consumed. This was to determine whether any differences in high calorie snack food intake resulted in an overall reduction in energy intake.

## Results

### Manipulation checks & demand awareness

All participants completed the experiment and were included in analyses. All participants were able to recall the message they had earlier read. Participants also categorised the three high calorie snack foods from the buffet as junk foods, with 123/129 identifying all three as junk foods. There was no evidence of demand awareness, in that participants were not aware of the true study aims. The cover story was widely believed. Of the 129 participants, only six mentioned that posters and eating study might have been linked. Removal of their data did not affect the results.

### Usual junk food intake

The two items measuring usual junk food intake were significantly correlated (r = 0.57, p < 0.05), so we averaged them to form an average measurement of usual junk food intake. A median split on the average measurement resulted in 67 participants being classed as high consumers of junk food (mean daily portions = 2.5, SD = 0.9) and 62 participants as being classed as low consumers of junk food (mean daily portions = 0.9, SD = 0.4).

### Baseline variables

The mean BMI of the sample was 23.2 (SD = 3.9, range = 16.1-41.4), which is within the healthy weight range of 18.5 – 24.9. The mean restraint score of the sample = 8.4 / 21, SD = 5.4, range = 0–21 (cronbach alpha = 0.88), suggesting that dieting tendencies were not high in the sample. The restraint scale ranges from 0–21 and the mean of this sample is similar to other studies sampling this population [22,25]. Mean baseline hunger score = 5.6 cm / 10.0 cm (SD = 2.2, range = 0.2-10.0). Hunger was marginally lower (p = 0.05) in the health message condition than the social norm message and control message conditions. See Table 1 for ANOVA results. Because of this we tested where baseline hunger was significantly correlated with junk food intake (r = 0.34, p <0.05). As baseline hunger was correlated with junk food intake we included baseline hunger as a covariate in subsequent analyses.

**Table 1:** Mean participant characteristics by condition

	**Social norm condition (n = 39)**	**Health condition (n = 48)**	**Control condition (n = 42)**	**Effect of condition**
**Baseline hunger** **(0–10 cm scale)**	5.9 (2.0)*	5.0 (2.2)	5.9 (2.5)*	p = 0.05
**BMI** **(metres/kg**^2^**)**	23.2 (4.8)	24.0 (3.9)	22.3 (2.9)	p = 0.12
**Restraint** **(0–21 scale)**	7.9 (5.6)	9.1 (4.8)	8.1 (5.7)	p = 0.49
**Age** **(yrs)**	23.7 (6.2)	22.0 (3.5)	22.0 (3.7)	p = 0.14

*compared to health condition, the social norm condition (p = 0.06) and control condition (p = 0.05) had higher baseline hunger.

### Food choice & intake

Participants tended to eat all of the food they selected (121 participants completely cleaned their plates). The remaining 6% only left a small amount of food, which totalled less than 20 grams of food, on average. The exact same pattern of results is obtained for food choice and food intake, so we only report analyses for food intake. See Table 2.

**Table 2:** Mean intake by condition

	**Social norm condition (n = 39)**	**Health condition (n = 48)**	**Control condition (n = 42)**
**High calorie snack food (grams)**	30.2 (20.9)*	23.4 (20.4)*	41.8 (37.7)
**Total snack (kcal)**	206.5 (121.9)^	164.5 (103.2)*	266.3 (210.1)
**Fruit and vegetables (grams)**	103.1 (74.3)	84.9 (58.4)	97. 0 (62.6)

*indicates significant difference at p < 0.05 to control condition, when adjusted for baseline hunger and usual junk food intake. ^ p = 0.06.

#### High calorie snack food intake

We first conducted a 2×3 ANCOVA on grams of high calorie snack, with baseline hunger as a covariate. As expected, baseline hunger significantly predicted grams of high calorie snack food [F(1, 122) = 13.1, p < 0.01, ηp^2^ = 0.10], whereby higher scores were associated with greater intake. Usual intake of junk food also predicted intake [F(1, 122) = 5.1, p =0.03, ηp^2^ = 0.04 ], whereby high usual consumers of junk ate more high calorie snack food than low consumers. Condition significantly predicted intake [F(2, 122) = 4.3, p < 0.02, ηp^2^ = 0.07]. However, there was no significant condition*usual intake of junk food interaction [F(2, 122) = 0.10, p = 0.91, ηp^2^ =0.001].

Next we wanted to follow up the effect of condition. As the results indicated that hunger was not balanced across conditions and usual junk food intake was associated with amount of high calorie snack food consumed, we wanted to control for these variables when assessing the effect of condition. To do this, we conducted linear regression analysis. We used dummy coding to assess the impact of both the social norm condition and health condition vs. the control condition on grams of high calorie snack food, whilst controlling for baseline hunger and usual junk food intake, by including them in the model. Both the social norm condition [B = − 11.5, p = 0.046] and the health condition [B = −14.7, p = 0.01] significantly predicted grams of junk food, whereby both were associated with reduced junk food consumption compared with the control condition. We also tested if the social norm and health condition differed in the effect they had on grams of junk food and they did not (p = 0.32). See Table 2.

### Additional analyses

#### Total calories consumed and fruit and vegetable intake

2×3 ANCOVA on total calories consumed, with baseline hunger as a covariate showed that baseline hunger significantly predicted total kcals consumed [F(1, 122) = 13.4, p < 0.01, ηp^2^ = 0.10]. Condition also significantly predicted total kcals consumed [F(2, 122) = 4.1, p = 0.02, ηp^2^ = 0.06] and usual intake of junk food approached significance [F(1, 122) = 3.3, p = 0.07, ηp^2^ = 0.03]. However, there was no significant condition*usual intake of junk food interaction [F(2, 122) = 0.10, p = 0.99, ηp^2^ < 0.001]. To follow up the significant main effect of condition, whilst accounting for usual intake of junk food and baseline hunger as continuous variables, we conducted the same linear regression analysis as for high calorie snack food intake. The social norm condition [B = − 59.1, p = 0.06] approached significance and the health condition [B = −82.7, p <0.01] significantly predicted total energy intake, whereby both were associated with decreased energy intake, compared with the control condition. The social norm and health conditions did not differ in the effect they had on total energy intake (p = 0.23).

In order to test whether condition differences in junk food intake resulted in differences in fruit and vegetable intake we also used the same analysis strategy to examine whether there were group differences for fruit and vegetable consumption. There was no main effect of condition in the 2×3 ANCOVA [F(2, 122) = .0.57, p = 0.57, ηp^2^ = 0.009] on grams of fruit and vegetables consumed. In conjunction with the results for total calories consumed this result suggests that participants in the social norm and health message conditions were not replacing junk food items with fruit and vegetables. See Table 2.

## Discussion

We examined whether messages about junk food intake would motivate individuals to decrease their consumption of high calorie snack food. Viewing messages outlining the health benefits of limiting junk food intake and that a social norm is to limit junk food intake was associated with less high calorie snack food intake than viewing a control message. There was no evidence that the social norm message had a greater effect than the health message. Compared with the control message, participants in the experimental conditions ate less high calorie snack food (32% on average, which equated to 70 fewer calories in a single snack session). These findings are promising, given that healthy eating interventions typically only promote modest changes to dietary behavior [10]. Although our measure of usual intake of junk food predicted the amount of high calorie snack food participants ate (usually high consumers ate more junk food), there was no evidence of an interaction between this measure and message type. Thus, the messages had similar effects on high calorie snack food intake across both high and low consumers of junk food. Total energy intake was also lower in the experimental conditions versus control condition, suggesting that the messages reduced junk food intake and there was little evidence of compensation or substitution with other food items.

Developing public health messages that encourage people to limit their consumption of high calorie foods is a priority, as the widespread availability and intake of these foods is thought to be a significant contributor to rises in adiposity [1,3]. Although much research has tested the types of interventions that could encourage people to increase their intake of fruit and vegetables, messages specifically targeting junk foods have received less attention [8,10]. The present study suggests two message types that may be effective. The use of social norm messages to encourage healthier eating is a novel concept but evidence has started to accumulate that they could be used to promote fruit and vegetable intake [20-22]. This is the first study to test the effectiveness of social norm message in reducing intake of junk food.

In a previous study, we observed that social norm messages about consumption of fruit and vegetables were associated with greater fruit and vegetable intake in individuals who were usually low consumers, but not high consumers. This is likely to be because high consumers were already adhering to the norm, whilst low consumers felt motivated to increase their intake to adhere to the presented norm [22]. See Schultz et al. for a discussion of how norms influence behaviour as a function of whether individuals believe they are adhering to a presented norm [23]. In the present study, we predicted that usual intake of junk food would moderate the effect of a social norm message on intake of high calorie snack food/junk food. There was no evidence for such moderation in the present study; lower junk food intake was associated with exposure to the experimental messages regardless of usual intake. One explanation for this finding may be that the low usual consumers of junk food did not perceive themselves to be adhering to the norm we presented. The norm message was that ‘others limit their junk food intake to 0 or 1 portion a day’ and very few participants reported normally consuming 0 portions a day. Thus, it could have been that all participants were motivated to adhere to the norm. Measuring participant perceptions of how their current behavior relates to a norm presented and testing the influence this has on junk food intake would allow for a more direct examination of this proposition in future studies.

Previously, we had also observed that social norm messages were more effective than health messages for promotion of fruit and vegetable intake [22]. A similar pattern of results has been found when measuring intentions to consume fruit and vegetables [21]. This was not the case in the present study as participants in both experimental conditions ate less high calorie snack food intake than the control condition. It is possible that health messages about fruit and vegetable intake are now commonplace [9,11], so the health implications of eating fruit and vegetables are already well understood. Conversely, messages about the health implications of consuming high amounts of junk food may not be as commonly understood in this population, so individuals may be less well informed. This suggestion is speculative, so evidence will be needed to confirm it. The message types assessed here are likely to bring about changes to eating behavior through different mechanisms (social motives vs. health motivates), so it would be interesting to examine whether an additive effect would be observed on combining messages. Examination of individual differences in responsiveness to message types would also be valuable. Health messages might be particularly influential on people with high health concern but they may have little impact on people with low health concern [26,27], whereas social norm messages may motivate both sets of people.

A strength of the present study was the inclusion of a control condition, in which participants were not exposed to food messages. This allowed us to make a direct comparison between intervention messages and a non-intervention message. In future studies it would be informative to test the effects of a message that instructs participants to reduce their junk food intake, without specific reference to health motives or social norms, as this would allow for a clearer examination of the importance of food-related message content. Eating behavior was measured shortly after exposure to messages, so although messages may have motivated participants to change their behavior in a healthy way, whether these changes would be maintained over longer periods requires examination. Social norm messages to promote other health behaviors have produced longer lasting effects, so longer term changes might occur [28]. As we tested participants in a lab environment and instructed them to read poster messages, it will be important in the future to examine whether these findings can be generalized to applied ‘real world’ settings. It will also be important to examine the effect of different social norm reference groups [29]. Here, young adult students participated and the social norm message was about what other *students* were eating. Thus, although this type of approach could be effective in university campus settings, further evidence will be needed to assess whether more encompassing norm messages would motivate healthy changes to dietary behavior and if effects would occur in other subsections of the population. That being said, some recent work has suggested messages about national eating norms can increase peoples intentions to eat healthily in the general population [21], so this deserves further attention. Examining whether social norms approaches could be used to promote healthy eating in young children also warrants attention, as Salvy and colleagues in particular have shown peers to have a substantial influence on snack food intake in a number of studies in this population [13,30].

The results of the present study suggest that participants did not compensate for their reduction in junk food by eating more of the fruit and vegetables that were also on offer. It may be the case that if other more palatable non-‘junk foods’ were available, participants would have compensated by eating these foods. Further work will need to clarify this. In line with other research examining social norm effects, we used self-report measures of dietary behavior [20,22]. Although we included a guided dietary recall and a measure of typical consumption, further work would benefit from measures less likely to be influenced by reporting bias.

The results from the present study show that messages about the health benefits and social norms surrounding ‘junk food’ intake are associated with reduced intake of high calorie snack food in young adults. The results also contribute to a new body of evidence that suggests social norm based messages can motivate people to eat more healthily.

## Competing interests

The authors declare that they have no competing interests.

## Authors’ contributions

ER, EH, JT, PA and SH conceived the study and participated in its design. EH and JT organised the co-ordination of the study. EH and ER conducted the research and analysed the data. ER, PA and SH were responsible for the first drafting of the manuscript and all authors approved the final manuscript.
